# A Porcine Animal Model for Early Meniscal Degeneration – Analysis of Histology, Gene Expression and Magnetic Resonance Imaging Six Months after Resection of the Anterior Cruciate Ligament

**DOI:** 10.1371/journal.pone.0159331

**Published:** 2016-07-19

**Authors:** Michael Kreinest, Gregor Reisig, Philipp Ströbel, Dietmar Dinter, Ulrike Attenberger, Peter Lipp, Markus Schwarz

**Affiliations:** 1 Department of Experimental Orthopedics and Trauma Surgery, Medical Faculty Mannheim, Heidelberg University, Mannheim, Germany; 2 Department of Pathology, University Medical Centre Göttingen, Göttingen, Germany; 3 Department of Radiology, Medical Faculty Mannheim, Heidelberg University, Mannheim, Germany; 4 Department of Molecular Cell Biology, University Medical Centre Homburg, Saarland University, Homburg/Saar, Germany; University Hospital of Modena and Reggio Emilia, ITALY

## Abstract

**Background/Objective:**

The menisci of the mammalian knee joint balance the incongruence between femoral condyle and tibial plateau and thus menisci absorb and distribute high loads. Degeneration processes of the menisci lead to pain syndromes in the knee joint. The origin of such degenerative processes on meniscal tissue is rarely understood and may be described best as an imbalance of anabolic and catabolic metabolism. A standardized animal model of meniscal degeneration is needed for further studies. The aim of the current study was to develop a porcine animal model with early meniscal degeneration.

**Material and Methods:**

Resection of the anterior cruciate ligament (ACLR) was performed on the left knee joints of eight Göttingen minipigs. A sham operation was carried out on the right knee joint. The grade of degeneration was determined 26 weeks after the operation using histology and magnetic resonance imaging (MRI). Furthermore, the expression of 14 genes which code for extracellular matrix proteins, catabolic matrix metalloproteinases and inflammation mediators were analyzed.

**Results:**

Degenerative changes were detected by a histological analysis of the medial meniscus after ACLR. These changes were not detected by MRI. In terms of their gene expression profile, these degenerated medial menisci showed a significantly increased expression of *COL1A1*.

**Conclusion:**

This paper describes a new animal model for early secondary meniscal degeneration in the Göttingen minipig. Histopathological evidence of the degenerative changes could be described. This early degenerative changes could not be seen by NMR imaging.

## Introduction

The menisci of the mammalian knee joint serve as shock absorbers and help to distribute the mechanical load on the tibial plateau. Each meniscus is divided into three parts: anterior horn (AH), posterior horn (PH) and Pars intermedia (PI) that is located in between the two horns. The ultrastructure of meniscal tissue consists of collagen fibers that are orientated circumferentially in the superficial layers and tangentially orientated in the layer in between [1]. The main protein in these fibers is type I collagen [2]. Other proteins involved in building meniscus’ extracellular matrix are collagen 2 and the proteoglycan aggrecan. The main cells in meniscal tissue are chondrocytes that, together with fibrochondrocytes and fibroblasts, form the extracellular matrix of fibrocartilage meniscal tissue [3]. Wimmer et al. [4] showed that chondrocytes also produce the lubricant lubricin that is also expressed in meniscal tissue [5, 6].

The degeneration of the menisci can be divided into primary and secondary degeneration. Both are morphologically defined as a disorder of the texture of the bradytrophic tissue. However, while primary degeneration is a disorder of texture which goes beyond the extent common for the patient’s age without any known clinical cause, secondary degeneration is clearly caused by diseases or injuries. Meniscal degeneration may be understood best as an unbalance between the anabolic and catabolic processes as it has already been demonstrated for articular cartilage [7]. Menisci which have been subject to degenerative changes show a hyperintensity in MRI and mucoid changes in the histological picture. The degeneration of menisci is rarely understood and currently being researched.

A detection of changes at the genome level in the expression profile of the chondrocytes in the meniscus should first be carried out. The anabolic genes in the extracellular matrix such as type I collagen and collagen-2 [7–11], the proteoglycan aggrecan [9, 10, 12–14] and the catabolic genes in the matrix metalloproteinases [7, 8, 12, 15] have been particularly well analyzed as marker genes of osteoarthritis in hyaline cartilage. In the fibrocartilage of the meniscus, the genome analyses are mostly limited to the presence of certain genes [16], so just a small number of typical markers such as C4d [17] were able to be described for this.

The analysis of the ultrastructure of degenerated menisci can show the effect that a change in expression profile has at the protein level. It is necessary to carry out histopathological tests to determine whether there is a link between a change in ultrastructure and a change in expression profile in the meniscus.

An animal model with standardized meniscal degeneration is required for the studies mentioned. An established animal model in osteoarthritis research is known as the Pond-Nuki model. The model, which was developed in dogs, shows a reliable development of osteoarthritis in the knee joint following an anterior cruciate ligament resection (ACLR) [18]. More recent studies using this model were able to show evidence of meniscal degeneration 24 weeks after ACLR by means of a 1.0 T MRI analysis [19]. However, this model was not evaluated towards meniscal degeneration, so to date no validated, standardized animal model with meniscal degeneration is described in the literature to our knowledge. However, since meniscal damage was also diagnosed by MRI in 90–98% of patients with an ACL insufficiency [20, 21] and MRI results are said to correlate with histological findings [22], ACLR appears to be a hopeful method of inducing secondary meniscal degeneration in an animal model.

Current literature refers to the pig as an appropriate animal model in biomedical research because of the similarity to human organism and metabolism [23, 24]. Even the structure of porcine collagen shows a high analogy with human collagen [25]. This was also shown for collagen in hyaline cartilage by Kääb et al. [26] by a comparison of different species. Since the development of the race of the Göttingen minipig [27], breeding has aimed to develop an adequate experimental animal with genetic uniformity, a friendly temperament, documented health status and a standardized, age-dependent weight.

The aim of the current study was therefore to induce meniscus degeneration in a standardized porcine animal model. Early degeneration should be analyzed using MRI, histology and gene expression profile.

## Materials and Methods

### Animals

The animal tests carried out were approved by the Karlsruhe Regional Council (ID: 35–9185.81/G-186/09). The experimental animals are pigs which have achieved full musculo-skeletal growth (Göttingen minipigs, Department of Animal Breeding and Genetics, University Göttingen). The mean age of the pigs was 24.8 ± 0.7 months (range: 23–25 months) and the mean body weight (BW) was 55.4 ± 9.4 g (range: 44–67 kg). In order to get a more homogenous group of knee joints, only female pigs were included in the current study since significant sex-specific differences in the porcine knee joint are reported [28]. The animals were cared for in accordance with the specifications of the supervising veterinarian. A visit was carried out twice a day for the first ten days after ACLR.

### Anesthesia

The Göttingen minipigs were given pre-medication by intramuscular injection (Azaperone, 4 mg/kg BW; Stresnil, Janssen, Beerse, Belgium. Ketamine, 10 mg/kg BW; Ketavet 10%, Vet Way, Elvington, Great Britain. Midazolam, 1 mg/kg BW; Midazolam-ratiopharm, Ratiopharm, Ulm, Germany). A venous cannula was inserted into the veins of the left and right ears. Propofol (Propofol-Lipuro, Braun, Melsungen, Germany) was then administered to induce anesthesia, enabling endotracheal intubation. The further preparations and the operation were carried out with balanced anesthesia with isoflurane, propofol and fentanyl.

The procedure set out above was initially carried out for anesthesia during MRI and the subsequent killing. In addition to this, a central venous catheter was inserted into the jugular vein and a transurethral bladder catheter was also inserted [29]. Purely intravenous anesthesia with pentobarbital was administered throughout the entire examination period. During the examination in the MRI machine, the animal was ventilated using a mechanical ventilator (Servo 300A, Maquet, Rastatt, Germany).

The pigs were killed under deep anesthetic by means of an intravenous injection of 20 ml saturated potassium chloride solution. Hypodynamic circulatory arrest documented in the electrocardiogram and by auscultation.

### Surgical procedure

The two rear limbs were washed three times (Softasept N, B. Braun, Melsungen, Germany) and covered sterile with the pigs on their backs stabilized by a vacuum mattress. Following intravenous application of cefazolin (2 g basocef, Deltaselect, Pfullingen, Germany), an incision in the skin of approximately seven cm was made in the left knee from the patella to the tuberositas tibiae. The joint was then opened medial to the patellar ligament and the patella was partly luxated. The ACL was then fixed by a clamp and cut at the distal end using a scalpel. To avoid spontaneous healing of the ACL after this transection, a proximal resection was additionally carried out using an electrical arthrosector and a Luer. Following successful rinsing with 60 ml of sterile 0.9% saline solution, sterile closure of the joint was carried out. Following the sterile closure of the wounds in layers, sterile bandages were applied to both hind legs.

The sham operation was then carried out on the right knee joint without any manipulation of the cruciate ligaments, menisci or articular cartilage. There was no randomization of the hind limbs for easy recognition of ACLR site and control site post-operatively when animals were kept in groups.

### MRI

Following a postoperative period of 26 weeks, MRI under intravenous anesthesia was carried out in a high-field magnetic resonance imaging machine (3 Tesla, Magnetom Trio, Siemens, Erlangen, Germany) with a 3 T CP Large Flex Coil (Siemens, Erlangen, Germany). The sagittal and coronary-angled proton density-weighted sequence "ProtonDensity fat-saturated" optimized from human knee imaging was applied. Slice thickness was 3 mm with 10% gap.

### MRI imaging scoring

It was not possible to blind the evaluation of the MRI images as the lack of the ACL was clearly identifiable. The MRI of the porcine knee joints were evaluated independently by two radiologists (DD, UA). An evaluation of AH, PH and PI of all menisci was carried out with regard to degenerative changes using the five-stage classification in accordance with Raunest et al. [30] (Table 1). The cruciate ligaments and the position of the tibia relative to the femur (phenomenon of the anterior drawer) were also assessed. If the evaluations of the two radiologists did not correspond with one another, consensus must be found by them assessing the images together.

**Table 1:** MRI-based classification of meniscal degeneration (adapted from [30]).

Grade	MRI results
0	homogeneous, weak signal intensity
I	local, punctiform increase in signal intensity in one layer with no connection to the surface of the meniscus
IIa	Some punctiform increases in signal intensity with no connection to the surface of the meniscus and no linear spread
IIb	Linear spread of the increase in signal intensity with no connection to the surface of the meniscus
III	Linear or irregular increase in signal intensity with contact with the surface of the meniscus or deformation/dislocation of fragments

### Preparation procedure of meniscal tissue

The animals were killed after MRI. The menisci were then isolated using a standardized preparation technique: after a clinical examination for swelling, effusion and the anterior drawer phenomenon, the knee joint opened up and the posterior cruciate ligament and the stump of the ACL exposed. Where still present, the ACL was severed. The femur and the tibia were then separated. The menisci were then carefully removed as a whole. The preparation described was carried out without touching the surfaces of the menisci or the hyaline articular cartilage in order to prevent contamination and the destruction of the surface structure. The equipment used for the preparation is kept free from RNAse by treatment with hydrogen peroxide [31].

The menisci are then divided for the various analysis methods. Samples were taken from the AH, PH and PI for histological evaluation (immediate fixing in 4% buffered formalin). Samples were also taken from the PI of the medial meniscus for immunohistological evaluation (immediate fixing in 4% buffered formalin) and for molecular biology evaluation (immediate asservation in liquid nitrogen).

### Histological procedures

Preparation and staining of the formalin fixed parts were carried out as described before [32].

### Immunohistochemistry

A control section with no primary antibodies for type I collagen (sc59772, anti-human from mice, Santa Cruz Biotechnology, Heidelberg, Germany) was incubated alone with each individual meniscus section. Following washing, blocking solution (BSA, Sigma-Aldrich) was applied to the sections (100 μl per section, 80 minutes). The primary type I collagen antibodies (dilution: 1:100; 100 μl per section) were then added and incubated for 70 minutes at room temperature. After washing, the biotinized secondary antibodies (Vectastain, Vector Laboratories; dilution: 1:100 in Tris20 buffered BSA) were added. The washing out of the secondary antibodies was then carried out and followed by a 30 minute period of incubation of the peroxidase bound to avidin. Following further washing, incubation with chromogenic diaminobenzidine (Vector Laboratories; 100 μl per section) is carried out. The preparation was then covered using a mounting medium (Eukitt, O. Kindler GmbH, Freiburg, Germany). Only a qualitative analysis was performed for immunohistochemistry.

### Semiquantitative histology scoring

The individual slides were analyzed by an experinced pathologist (PS) using a light microscope (Axioskop, Zeiss, Jena, Deutschland) and graded using a four-stage meniscus score [32] based on the HE-staining described above. Major criteria are listed in Table 2. Grading of degeneration was based on the pathologist’s decision what bundle of criteria (Table 2) fits most to the slides. To reduce bias of this method, the pathologist was blinded towards the treatment group (ACLR vs. Control).

**Table 2:** HE-staining-based four-stage scoring system for meniscal degeneration [32].

Grade	Criteria
0	Homogenous eosinophilic matrix staining No reduced cellularity Uniform morphology of chondrocytes No matrix clefts
1	Basophilic matrix staining Slight reduction of cellularity or small areas with reduced cellularity Small matrix clefts Minor polymorphism of chondrocytes Increased banding of fibrocartilage Possible accumulations of fat, minor chondroid metaplasia
2	Moderate basophilic matrix staining Obvious reduction of cellularity or multiple areas with reduced cellularity Matrix clefts Polymorphism of chondrocytes Banding of fibro-cartilage Fat accumulations
3	Marked basophilic matrix staining Paucicellular, multiple obvious matrix clefts Marked polymorphism of chondrocytes and banding of fibrocartilage Fat accumulation

### Quantitative real time PCR

Processing of the PI of the medial menisci were carried out as described before [32]. The specific primers and annealing temperatures are found in Table 3. The housekeeping gene ß-actin was chosen as reference, since its expression is said to be very stable in most porcine tissues [33]. Gene expression was evaluated as amount of RNA normalized to amount of *β-ACTIN* (normalized amount ± standard deviation [%]).

**Table 3:** Primer sequence, annealing temperatures (AT) and PCR product size for the different genes.

Gene		Primer sequence	AT	PCR product size [bp]
Aggrecan	*ACAN*	F: TTCCCTGAGGCCGAGAAC	56°C	194
		R: GGGCGGTAATGGAACACAAC		
β-Actin	*ACTB*	F: CAAGGAGAAGCTCTGCTACG	56°C	245
		R: AGAGGTCCTTCCTGATGTCC		
Decorin	*DCN*	F: GCCAGAGAAAATGCCCAAAAC	56°C	117
		R: GTGCCAAGTTCTACGACGAT		
Interleukin-1 beta	*IL1B1*	F: CAGCCATGGCCATAGTACCT	57°C	216
		R: CCACGATGACAGACACCATC		
Interleukin-8	*IL8*	F: TGCAGCTTCATGGACCAG	57°C	355
		R: TGTTGCTTCTCAGTTCTCTTC		
Type I collagen	*COL1A1*	F: CCAACAAGGCCAAGAAGAAG	54°C	64
		R: ATGGTACCTGAGGCCGTTCT		
Collagen 2	*COL2A1*	F: CACGGATGGTCCCAAAGG	54°C	102
		R: ATACCAGCAGCTCCCCTCT		
Lubricin	*PRG4*	F: AGAAAACCCGATGGCTATGA	56°C	150
		R: TCGCCCATCAGTCTAAGGAC		
MMP-2	*MMP2*	F: GGCTTGTCACGTGGTGTCACT	58°C	68
		R: ATCCGCGGCGAGATCTTCT		
MMP-3	*MMP3*	F: AATGATCACTTTTGCAGTTCGAGAA	56°C	76
		R: GGCATGAGCCAAAACTTTTCC		
MMP-8	*MMP8*	F: CATTTTGATGCAGAAGAAATATGG	52°C	70
		R: CATGAGCAGCAACAAGAAACA		
MMP-9	*MMP9*	F: GAAGCTTTAGAGCCGGTTCCA	58°C	96
		R: GGCAGCTGGCAGAGGAATATC		
MMP-13	*MMP13*	F: TTGATGATGATGAAACCTGGA	52°C	69
		R: ACTCATGGGCAGCAACAAG		
SOX-9	*SOX9*	F: CCGGTGCGCGTCAAC	56°C	119
		R: TGCAGGTGCGGGTACTGAT		
VEGF	*VEGFA*	F: GAGACCAGAAACCCCACGAA	57°C	138
		R: GCACACAGGACGGCTTGAA		

bp = base pairs

### Sample size calculation

The grade of meniscal degeneration determined using the MRI was used as the main criteria in the animal tests. This was determined using a five-stage scoring system (Table 1). The meniscus was deemed to be degenerated from grade IIa. There was therefore a difference of two units compared to a healthy meniscus (grade 0). In order to receive an estimation of the power of 80% with a standard significance level of 5% [34], a case number of n = 8 animals was calculated using a unilateral t-test.

### Statistical analysis

Statistical analyses were carried out using the software SAS 9.1 for MS Windows (Microsoft Corporation, Redmond, USA). The ACLR group and the control group were deemed to be independent. Group-wise comparisons were made using the U-test by Mann-Whitney. Since multiple genes were tested, false discovery rate (FDR) was calculated according to the Benjamini and Hochberg method [35]. Significance threshold was set at p < 0.05 and FDR < 0.1. Dot plots show median as well as 25% and 75% quartile.

## Results

### Operative treatment

ACLR and sham operation was able to be carried out completely on the knee joints of eight fully grown Göttingen minipigs.

### Postoperative care

Any attempts to rest the hind legs postoperatively were viewed as an expression of pain and adequate analgesia (Caprofen, Rimadyl, Pfitzer, Berlin, Germany) was provided immediately. During the second week after the operations, the animals started to put weight on both hind legs. While full weight was put on the rear right leg (sham operation), there was an avoidance to put full weight on the left. It was not always possible to attribute if the avoidance was due to the experience of pain or due to the presence of the instability of the left knee joint after ACLR. Where there was any doubt, the animal was given analgesics. From the fourth week after the operation, the animals started to put increased weight on the rear left leg and in some cases to put their full weight on this leg.

According to the ARRIVE guidelines for reporting animal research [36] we have to report that one Göttingen minipig that was included in a pilot study of three animals (data not shown) has to be killed due to an infection in the left knee joint after ACLR.

### MRI

**Fig 1:**
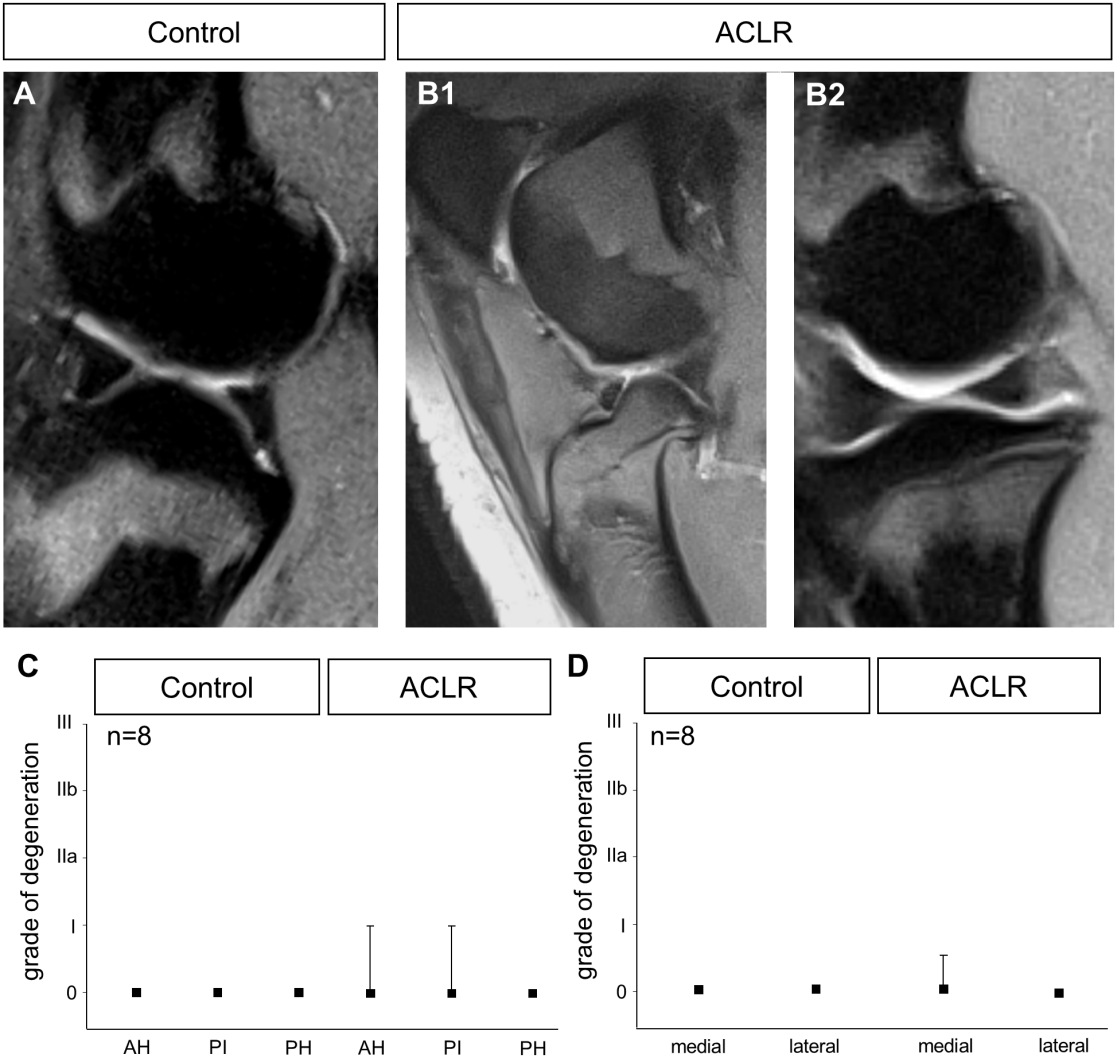
MRI of the knee joint in the control group and after ACLR. In the sagittal cross section of a control knee (A), the medial meniscus is homogenously black. The femur and tibia are on top of one another. In the knee joint after ACLR, the lack of the ACL caused the tibia to be moved forwards (B1). However, the menisci are still homogeneously black (B2). There is no evidence of degeneration in AH, PI and PH of the medial menisci (C). There is also no evidence of degeneration in the lateral menisci (D).

After a period of 26 weeks, MRI was carried out on the Göttingen minipigs. The independent evaluation of the MRI by two radiologists showed a high grade of correlation between the assessments towards the classification of the degeneration. Consensus had to be reached in three cases. The analysis of the MRI showed that ACLR was successful in all left knee joints. In individual cases, this caused a displacement of the tibia in an anterior direction (anterior drawer phenomenon, Fig 1B1). In all of the knee joints in both groups, the posterior cruciate ligament was intact.

In the magnetic resonance images, both the menisci in the knee joints in the control group (Fig 1A) and the menisci in the knee joints after ACLR (Fig 1B2) were homogeneously black. No clear change in signal intensity, which could be interpreted as a symptom of degeneration was observed. Following an evaluation of all medial menisci, there was no significant degeneration of AH, PH or PI (Fig 1C) of the menisci after ACLR. The overall comparison of all of the menisci in both groups showed that there was no evidence of significant degeneration in the lateral or in the medial meniscus after ACLR (Fig 1D).

### Macroscopic findings

Once the MRI had been carried out, the animals were killed under deep anesthetic. All of the animals showed pronounced instability on the left hand side (anterior drawer phenomenon). Instability or subluxation of patellae was not evident in any animal. After this examination, the knee joints were quickly prepared. All of the posterior cruciate ligaments were intact, with no exceptions. All of the ACL were also intact on the right side (control). All of the ACL were missing in the left knee joint. The macroscopical assessment of the tibial and condylar cartilage of the knee joints showed variable states of hyaline cartilage alteration (as described elsewhere). No evidence of the macroscopic mucoidal staining of the menisci described by Ferrer-Roca et al. [37] was able to be found.

### Histological scoring

**Fig 2:**
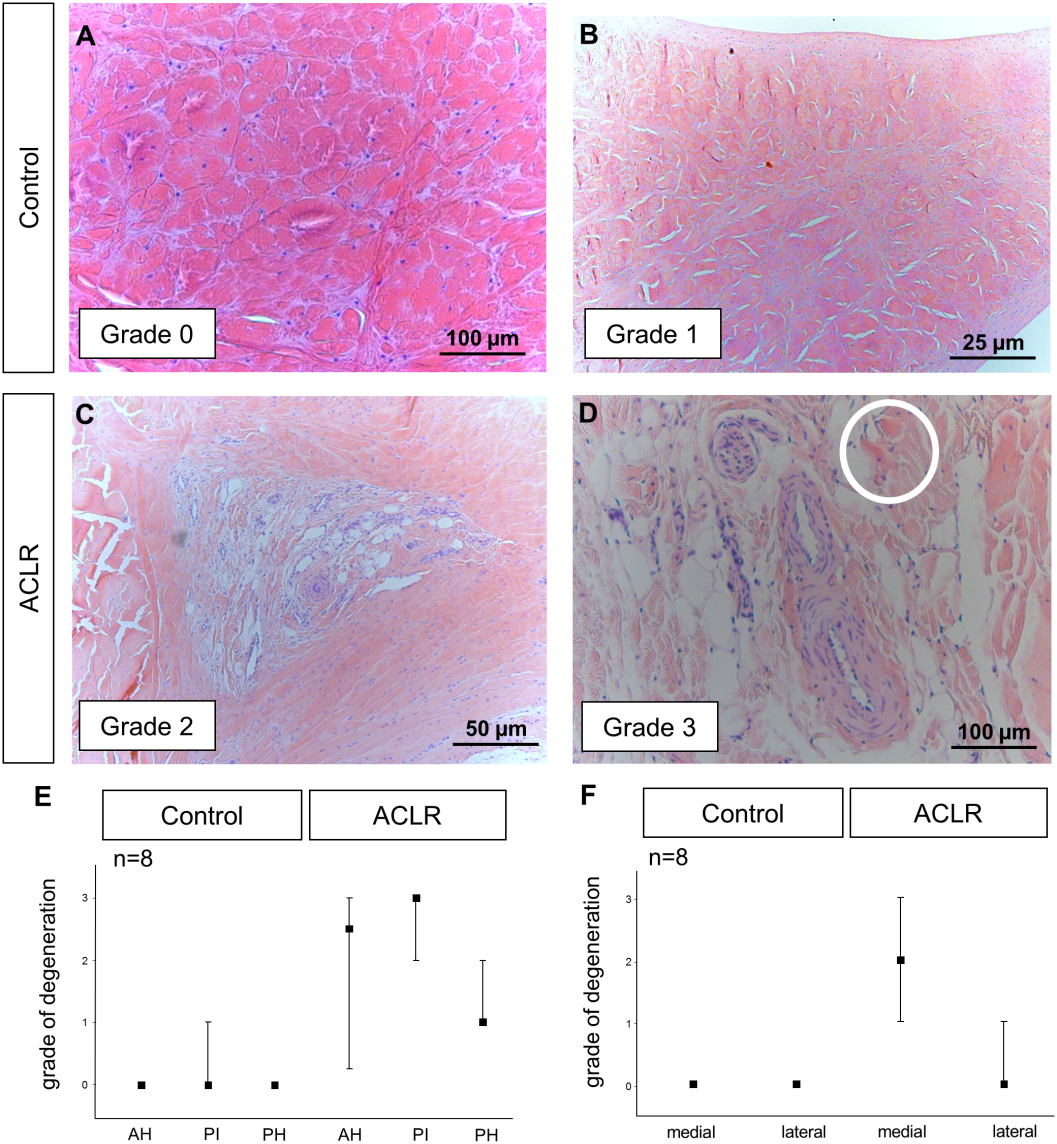
Histological evaluation of the degeneration in the PI of menisci in the control (A, B) and ACLR (C, D) group. A four step scoring was performed using hematoxylin-eosin staining. In case of homogeneously stained matrix with balanced cell distribution (A) degeneration was graded O. If additional matrix clefts occurred, degeneration was graded 1 (B). Degenerative changes such as moderate basophilic (blue) matrix staining in accordance with matrix clefts have been graded 2 (C). Additional mucoid changes (circle in D) and the separation of the fibrocartilage represent third grade degeneration (D). The grade of degeneration in AH, PH and PI of all medial menisci has been determined (E). Clear degeneration was only able to be observed in the medial menisci after ACLR (F).

Fig 2A shows a representative sectional preparation from the PI of a medial meniscus of the control group. These slides could be best characterized by a homogenous eosinophilic matrix staining with normal cellularity (Grade 0). In some cases, additional small matrix clefts were also detected in sectional preparations from the PI of medial menisci of the control group as shown in Fig 2B (Grade 1). No signs of high-grade degeneration were able to be observed in either AH, PH or PI of the medial menisci of the control group (Fig 2E). The medial menisci after ACLR showed clear signs of degeneration such as moderate basophilic matrix staining (blue staining of the preparation; Fig 2C) and matrix clefts in the PI (Fig 2C) but also in the AH and PH (Grade 2). In some slides of meniscal tissue after ACLR additional pronounced mucoid changes which are characterized by less stained and overstained areas (circle in Fig 2D) in accordance with separation and the formation of tears and multiple obvious matrix clefts have been seen mainly in the PI (Fig 2D) but also in the AH (Grade 3). Other criteria for high-grade degeneration that have been described previously [38, 39] such as polymorphism of chondrocytes and fat accumulations were rare in the menisci of the current study. The lateral meniscus after ACLR showed none of the degenerative changes of this type.

Degeneration was significantly increased in the AH (p < 0.01), the PI (p < 0.001) and the PH (p < 0.01) of the medial menisci after ACLR compared to the corresponding areas of the medial menisci of the control group (Fig 2E). The overall comparison of the menisci which includes the average scores from AH, PH and PI showed that only the medial meniscus after ACLR showed a significant grade of degeneration (p < 0.001; Fig 2F).

### Analysis of gene expression

All gene expressions were normalized to the expression of the housekeeping-gene *β-ACTIN*. The amplification efficiency of all qRT-PCR reactions performed was 90.5 to 104.8% as calculated from the slope of the standard curves.

Messenger RNA encoding for *COL1A1* was expressed significantly more in the medial menisci after ACLR (Table 4). *Il1B1* expression was virtually absent in both groups. Messenger RNA encoding for Lubricin was only detected in very small amounts and only in 6 samples. All of the other genes analyzed showed no significant differences in gene expression after ACLR compared to the control group (Table 4).

**Table 4:** Results of the qRT-PCR. The normalized expression of medial menisci in the control and ACLR group is compared and the changes in expression are described.

Gene	Normalized gene expression [%]		t-Test	FDR
	Control (n = 8)	ACLR (n = 8)		
*COL1A1*	0.48 ± 0.28	1.21 ± 0.82	p < 0.05	0.08
*COL2A1*	0.80 ± 0.82	0.44 ± 0.23	n. s.	n. s.
*ACAN*	40.72 ± 59.54	17.39 ± 11.74	n. s.	n. s.
*SOX9*	0.04 ± 0.03	0.47 ± 0.95	n. s.	n. s.
*Il1B1*	not detectable	not detectable	-	-
*IL8*	0.08 ± 0.14	0.03 ± 0.02	n. s.	n. s.
*MMP2*	2.65 ± 1.15	4.12 ± 1.88	n. s.	n. s.
*MMP3*	5.67 ± 4.04	6.41 ± 4.36	n. s.	n. s.
*MMP8*	0.49 ± 0.22	0.68 ± 0.78	n. s.	n. s.
*MMP9*	0.08 ± 0.21	0.01 ± 0.02	n. s.	n. s.
*MMP13*	3.39 ± 6.61	3.11 ± 5.61	n. s.	n. s.
*VEGFA*	1.04 ± 0.80	0.76 ± 0.59	n. s.	n. s.
*PRG4*	1.42 ± 1.25 *	0.56 ± 0.70 *	-	-
*DCN*	76.95 ± 21.54	161.85 ± 148.87	n. s.	n. s.

* *PRG4* was only detected in n = 6 samples

### Immunohistochemistry

**Fig 3:**
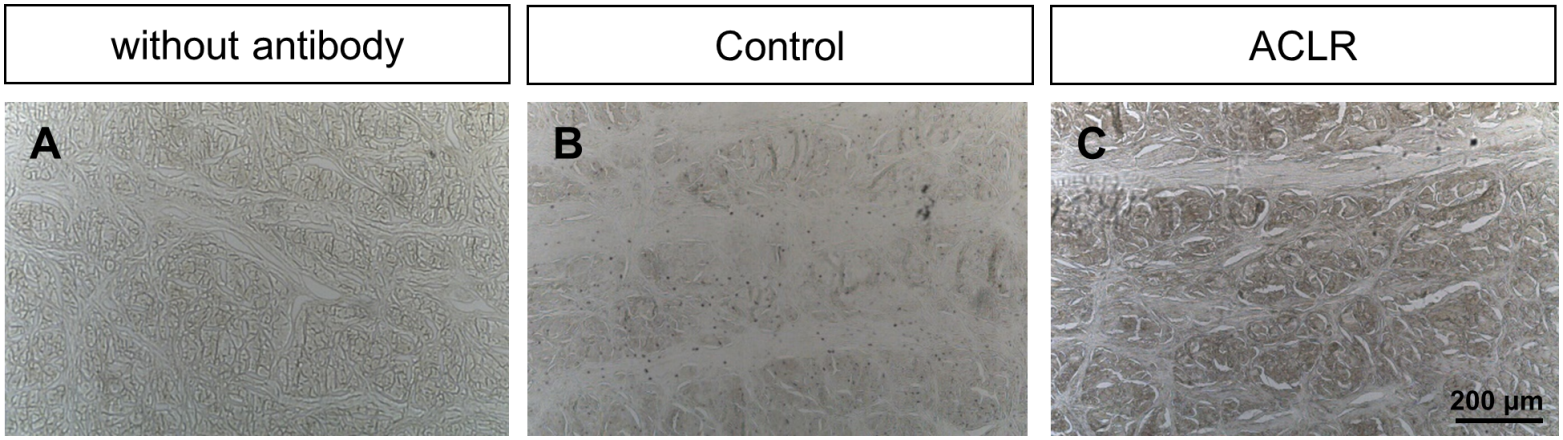
Qualitative immunohistochemical analysis of type I collagen in the PI. No staining without antibody (A), weak staining in the control group and more intense staining after ACLR (C).

The section preparations from the control group showed weak to moderate staining (Fig 3B). The staining of the section preparations from the menisci after ACLR showed more intense staining (Fig 3C).

## Discussion

The present study showed that degenerative processes are present in porcine menisci 26 weeks after ACLR. The HE staining proved destruction of the collagen network indicating that degeneration is mainly driven by structural changes of the matrix’ integrity. On the other hand, no degeneration was detected in MRI. Even gene expression showed only a significant increase for *COL1A1*. Genes said to play an important role in degeneration did not change their expression in this study. The degenerative processes detected in this study may therefore be characterized as early degeneration.

### Animal model

To date, neither pigs in general nor the Göttingen minipig in particular have been described in meniscal degeneration research. The adapted Pond-Nuki model, in the meaning of an isolated ACLR was able to be transferred from dogs [19] to the female Göttingen minipig in this study. From human medicine, it is known that an insufficiency of the ACL can lead to meniscal damage [20, 21]. In the current study, we were able to trigger early secondary meniscal degeneration which was confirmed by histological evidence. The cause of the occurrence of this meniscal degeneration could be the following mechanism: Due to the lack of the knee joint’ stabilizer [40], ACLR leads to instability in the knee joint, meaning that each time weight is put on the limb (approx. 40% of the body weight is on the rear limbs in quadrupeds) a physiological force is exerted on the menisci. Since 60-80% of the weight acts on the medial part of the knee joint [40], this could lead to the secondary degeneration of the medial meniscus described. The fact that fibrocartilage also showed degenerative changes caused by compression loads was also demonstrated on the intervertebral discs [41].

Differences in knee joint anatomy of pigs compared to dogs such as tighter joint space and pronounced menisci may contribute to the development of meniscal degeneration. Further studies must show if these morphological differences contribute to the lack of MRI detection of degeneration in the porcine model compared to the dog model six months after ACLR.

### MRI

In dogs, evidence of degenerative changes to the medial meniscus following ACLR and a postoperative period of 24 weeks was able to be provided using MRI [19]. The histopathological evaluation of meniscal degeneration carried out for the current study also showed degenerative changes to the medial meniscus. However, no evidence of this could be found by MRI. Studies comparing the sensitivity of the detection of meniscal damage using MRI and histopathology show a similarly sensitive evaluation result [22]. But mostly, it is only the detection of meniscal lesions which is assessed in these studies [42] and not the detection of degenerative changes. The use of MRI to detect isolated meniscal degeneration is experimental and is mostly indirect [43]. A conceivable cause of errors could be the so-called phenomenon of the magic angle [44]. In the current study, however, the results of the MRI did not correspond to the histopathological reference evaluations.

### Histology

For histological evaluation a published score for human meniscal tissue degeneration [30, 39] had to be adapted as described before [32] since there is, to our knowledge, no other published scoring system for meniscal degeneration of porcine tissue. The lateral menisci from both knee joints and the medial meniscus from the right knee joint (control) showed mainly normal meniscal tissue that is homogenous matrix staining and high cellularity with uniform morphology of the chondrocytes. The overall degeneration grade of the medial menisci isolated from the left knee joint was significantly increased. Most of the criteria for grading meniscal degeneration concern structural changes of the meniscal tissue.

### Gene expression

Differences in the meniscal extracellular matrix as they were seen in the histological analysis could be caused by changes in gene expression. In order to look for differences on the gene expression level a qRT-PCR on 14 selected genes was carried out. The selection of the genes analyzed was mainly based on marker genes for degeneration of hyaline cartilage in the knee joint [7, 9] or fibrocartilage in vertebral disc tissue [45], since literature on marker genes for meniscal tissue degeneration is rare. Since a pilot study (data not shown) did not show any differences in terms of the gene expression profile depending on the intrameniscal localization (AH vs. PH vs. PI), the analysis of the gene expression was only carried out from the PI of the isolated menisci. All of the genes investigated are described as marker genes for degeneration in hyaline articular cartilage [7–9, 15] and evidence of them was found in the meniscus [16].

Genes in the first group code for extracellular matrix proteins (*COL1A1* and *COL2*) and for matrix-associated proteins such as the proteoglycans aggrecan (*ACAN*), decorin (*DCN*) and lubricin (*PRG4*). Type I Collagen is the main structural protein of meniscal tissue [11] and shows a significant difference in gene expression in the present study, as already described for the comparable animal model in dogs [46]. If the increase in gene expression also leads to an increase in the quantity of the protein type I collagen in the meniscus tissue after ACLR must be analyzed in further studies since qualitative immunohistochemical analysis as performed in the current study cannot prove a change in protein expression level. Lubricin, also known as proteoglycan-4, is produced in chondrocytes [4] and is found on the tibial and femoral meniscal surface [5] as well as in deeper tissue areas [6]. *PRG4* is known to change its expression in the case of acute cartilage tears [47] as well as in the presence of chronic degenerative changes of hyaline cartilage, such as osteoarthritis [5]. In the present study, gene expression of *PRG4* was detected in only six samples. Further statistical analysis was therefore not possible. The remaining genes which were investigated, coding for structural proteins, matrix metalloproteinases or inflammation mediators, showed no significant expression changes. Missing changes in gene expression profile may be explained by the origin of the degeneration of the meniscal tissue. Most degenerative changes were driven by a loss of structural integrity of the matrix indicating a mechanical cause. Changes in gene expression may only be seen after alteration of chondrocytes that were rare in the current study.

In general, there were large standard deviations for the genes analyzed. It is known that there are significant differences between individual chondrocytes from menisci which have been subject to degenerative changes [48] and that even chondrocytes in cell culture constantly change their expression profile [49]. Nevertheless, this must lead to a very critically interpretation of the qRT-PCR results as false negative results may occur due to the broad distribution of data. The heterogeneity of the chondrocytes which generally occurs [6] can contribute to this high interindividual variability. Further studies are needed to determine whether type I collagen can be used as a marker gene for early meniscal degeneration even if MRI-based evidence is absent. Furthermore, type I collagen gene expression may vary at different time points of degeneration process due to increased cell death if degeneration deteriorates

### Limitations

The current study is limited to some extent. Meniscal degenerative processes after ACLR were compared to menisci isolated from a sham operated knee joint. A normal control group including menisci from non-operated knee joints is missing and should be provided in further studies. Furthermore, degeneration has not been detected by all analyzing methods used in the current study. Results of gene expression analysis should be interpreted reluctant since there were large standard deviations for the genes analyzed. Immunohistochemical analysis is only descriptive since quantification has not been performed.

In summary, in the current study it was possible to describe a new animal model for secondary meniscal degeneration in the female Göttingen minipig. Histopathological evidence of the degenerative changes was able to be found. It was not possible to find evidence of the degenerative changes using MRI procedures. Many genes known as degeneration markers showed no typical changes in their expression. The changes described appear to relate to an early stage of meniscal degeneration. Further studies are needed to determine whether extending the period of time after ACLR will provide evidence of significantly more pronounced degeneration.
